# Application of 3D Printing Technology in Sensor Development for Water Quality Monitoring

**DOI:** 10.3390/s23052366

**Published:** 2023-02-21

**Authors:** Yifan Sun, Dunzhu Li, Yunhong Shi, Zeena Wang, Saviour I. Okeke, Luming Yang, Wen Zhang, Zihan Zhang, Yanqi Shi, Liwen Xiao

**Affiliations:** 1 Department of Civil, Structural and Environmental Engineering, Trinity College Dublin, D02 PN40 Dublin, Ireland; 2 TrinityHaus, Trinity College Dublin, D02 PN40 Dublin, Ireland

**Keywords:** 3D printing, sensor, water quality monitoring, fabrication material, floating platform, sensing electrode

## Abstract

The development of sensors for water quality monitoring is crucial to protect water quality, aquatic biota and human health. Traditional sensor manufacturing methods have significant drawbacks, such as low fabrication freedom, limited material choice and expensive manufacturing cost. As a possible alternative method, 3D printing technologies are increasingly popular in sensor development due to their high versatility, fast fabrication/modification, powerful processing of different materials and ease of incorporation with other sensor systems. Surprisingly, a systematic review examining the application of 3D printing technology in water monitoring sensors has not yet been conducted. Here, we summarized the development history, market share and advantages/disadvantages of typical 3D printing techniques. Specifically focused on the 3D-printed sensor for water quality monitoring, we then reviewed the applications of 3D printing in the development of sensors’ supporting platform, cell, sensing electrode as well as all-3D-printed sensors. The fabrication materials and processing, and the sensor’s performances regarding detected parameters, response time and detection limit/sensitivity, were also compared and analyzed. Finally, the current drawbacks of 3D-printed water sensors and potential directions for future study were discussed. This review will substantially promote the understanding of 3D printing technology used in water sensor development and benefit the protection of water resources.

## 1. Introduction

Globally, the scarcity of water resources is one of the major challenges for aquatic life and human health [1,2,3]. The mismanagement of water resources and human-related water pollution have a substantial adverse effect on water shortages [1,4,5]. According to Human Development Reports 2020 published by the United Nations, 80% of wastewater is discharged into the environment without treatment [6,7]. The pathogen and organic pollutant levels in rivers in Africa, Asia and Latin America rose by more than 50% from 1990 to 2010 [6,7]. To tackle this pollution, many governments and organizations have enacted environmental laws and regulations to regulate pollutant discharge, manage waste disposal and reduce environmental pollution, especially the pollution level in water bodies, Zhao, et al. [8]. 

The development and application of cost-effective sensors are crucial to understanding water pollution levels, assessing the efficiency of relevant environmental management and policy and raising an early alert for potential water contamination in the future, which can effectively protect water quality, aquatic biota and human health [9,10]. There are some general requirements for water analysis sensors, including low cost, high sensitivity, good reliability and a long lifetime [10,11]. Traditional sensor manufacturing methods have significant drawbacks, such as low fabrication freedom, limited material choice and high manufacturing cost [12]. Additionally, the trend of miniaturization and automation, as well as the importance of green chemistry, have brought new challenges to the manufacturing and development of water analysis sensors [13,14]. 

For instance, to continuously monitor water quality, the sensor system usually needs a floating platform that will not capsize during strong water turbulence and powerful wind [15]. Additionally, the cell-encapsulating sensing component needs to be resistant to potential interference factors, such as water flow rate, ambient temperature and strong UV-induced degradation/damage [16]. More importantly, the sensing component, such as the electrode, should maintain high flexibility and sensitivity to make good contact with water samples and detect targeted pollutants with high accuracy [17]. It is also evident that the re-design and modification of the sensor’s configuration are highly important to improve the detection performance and meet the requirements to monitor emerging water pollutants [18,19]. Although traditional methods may achieve these goals with high cost, 3D printing technology has been considered as a more promising solution to tackle these challenges. It is a powerful tool that allows rapid/precise fabrication of sophisticated structures and devices with complex geometry, cost-effective modification, powerful processing of different materials, ease of incorporation with other sensor systems and minimum human interventions [14,20,21]. 

Given these advantages, 3D printing technology has been increasingly applied to the development of sensors to monitor and analyze water quality in the last 10 years. It can be used to develop a complicated floating platform to support the main parts of the water sensor [15], create a novel cell to prevent interference and effectively mix water samples [11] and produce sensitive sensing elements such as flexible electrodes [17]. Surprisingly, a systematic review examining the application of 3D printing technology in water monitoring sensors has not yet been conducted. This hinders the selection and application of 3D-printed sensors for water quality monitoring. Here, we have summarized the development history, market share and advantages/disadvantages of typical 3D printing techniques. Specifically focused on the 3D-printed sensors for water quality monitoring, we have reviewed the applications of 3D printing in the development of sensors’ supporting platform, cell, sensing electrodes and all-3D-printed sensors. The fabrication materials and processing, and the sensor’s performances regarding detected parameters, response time and detection limit/sensitivity, have also been compared and analyzed. Finally, the current drawbacks of 3D-printed water sensors and potential directions for future study are discussed. This review will substantially promote the understanding of 3D printing technology used in water sensor development and benefit the protection of water resources.

## 2. The Development and Market Share of 3D Printing Technology

**Figure 1:**
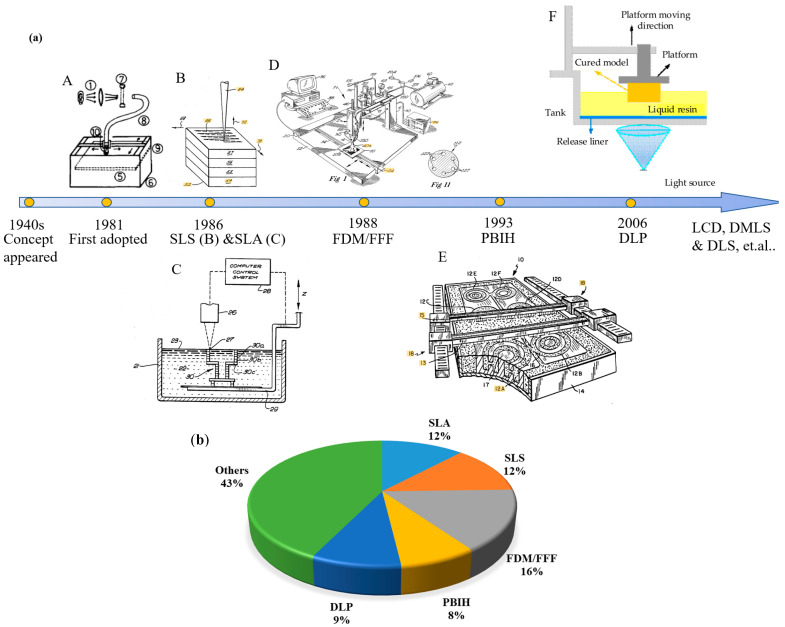
(**a**) Brief history of 3D printing development. **A**: The diagram of the first adopted 3D-printing technology [23], **B**: The diagram of SLA [25], **C**: The diagram of SLS [29], **D**: The diagram of FFF/FDM [32], **E**: The diagram of PBIH [33], **F**: The diagram of DLP [34]. All cited figures in this paper were reproduced with permission. (**b**) The most used 3D printing technologies worldwide in 2021, data were obtained from [28].

Over time, 3D printing (or additive manufacturing) has been developing a variety of technologies (Figure 1) and has been adapted to work with a broad range of materials and applications [22]. The concept of additive manufacturing appeared in the 1940s and was first adopted by Hideo Kodama in 1981 [23] (Figure 1(aA)), while Charles Hull developed the first commercial 3D printing technology, which was known as stereolithography (SLA) (Figure 1(aB)) [24,25]. SLA uses UV light (or electron beam) to convert monomers (mainly acrylic or epoxy-based) to polymer chains to create solidified layers that will hold the subsequent layers. SLA can print high-quality parts with a fine resolution (as low as 10 μm) but has a low printing speed and limited materials’ choice [26,27]. Nowadays, SLA still has wide applications. In 2021, 11.9% of global users employed SLA for their applications (Figure 1b) [28]. 

The selective laser sintering (SLS) technique was developed and patented by Dr. Carl Deckard at the University of Texas at Austin [29] in 1986 (Figure 1(aC)). SLS is a powder processing technique. It uses a laser beam to sinter powder together by heating and then the processing of the next layer starts [27,30]. SLS can use a wide range of materials and does not need a supporting base; however, it requires the unit to be a large physical size and has high power consumption and a poor surface finish [31]. After its development, SLS has gained wide application, and in 2021, it was the second most widely used 3D printing technology (12.6% market share of global 3D printing users) and the most common in external service (Figure 1b) [28].

Two years after the invention of SLA, in 1988, fused deposition modelling (FDM), also known as fused filament fabrication (FFF) (Figure 1(aD)), was developed by S. Scott Crump, co-founder of 3D printer manufacturer Stratasys, Ltd. (1989, Minnesota, America) [31]. FDM/FFF printers work by controlled extrusion of thermoplastic filaments, which melt into a semi-liquid state at the nozzle and are extruded layer by layer and then solidify into final parts [27]. Currently, FDM/FFF is the most widely used 3D printing technology, with a 15.7% market share of global 3D printing users (Figure 1b) [28], due to its low cost, high printing speed, simplicity and capacity for depositing multiple materials simultaneously [26,35]. However, the layer-by-layer appearance and the materials that must be composite in a filament form are the main drawbacks and challenges for FDM/FFF [27,36].

Powder bed and inkjet head 3D printing (PBIH) (Figure 1(aE)), also known as binder jet 3D printing, was developed at the Massachusetts Institute of Technology and patented in 1993 [33,37]. As a powder-based layered printing process, the main advantage of PBIH is its multi-material capability—any polymer materials in powder state may be printed by this technology [37]. Poor printing resolution is the main disadvantage of this technology [27]. In 2021, 7.9% of global users employed PBIH for their applications (Figure 1b) [28].

Digital light processing (DLP) (Figure 1(aF)) was not initially developed for 3D printing, but it was applied to 3D printers in 2006 [34,38]. DLP is very similar to SLA, instead of using a laser, DLP uses a digital light projector to cure the resin. DLP is a super-fast form of 3D printing but only can use photo-curable resins as print material [24,39]. Currently, there are 9.1% of global users who have chosen DLP for their applications (Figure 1b) [28].

Recently, several new techniques have been developed for 3D printing, such as the polyjet [40], the direct metal laser sintering (DMLS) [41] and the digital light synthesis (DLS). Evidently, the development and the relevant applications of 3D printing technology are still ongoing and further improvement is expected.

## 3. Applications of 3D Printing in Sensors’ Development

**Figure 2:**
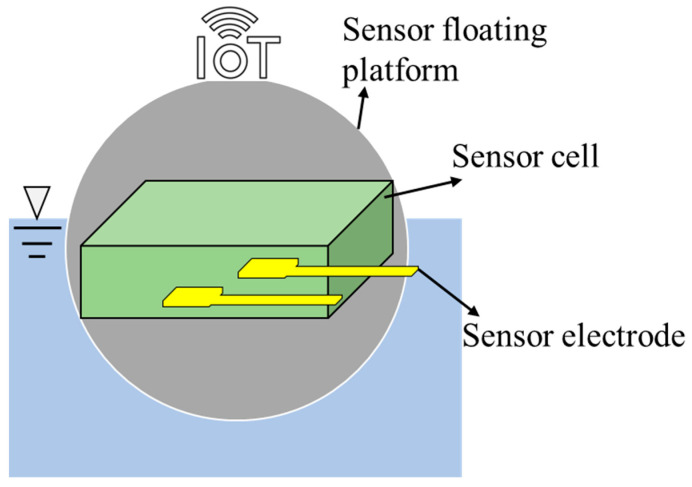
Schematic diagram of the typical sensor for water quality analysis. Three-dimensional printing can be employed to fabricate the sensor’s supporting platform, cell and/or electrode.

To monitor water quality, a typical sensor consists of three components: the supporting platform, the sensor cell to contain sensing elements and water sample and the sensing elements such as the electrode to detect the targeted pollutants in the water sample (shown in Figure 2). Three-dimensional printing has been successfully employed to fabricate not only individual components but also the whole sensors for monitoring water quality such as the hardness, turbidity and toxicants of heavy metals. To identify the impactful studies related to this topic, the online databases (Web of Science and Google Scholar, both accessed on 10 February 2023) were searched using the following key terms: (3D print or additive manufacturing) and (sensor or monitor) and (water or aquatic). The search obtained around 450 relevant results, which were further screened by reviewing the abstracts. Finally, 39 papers were identified as the key papers, which were carefully reviewed (full paper) to understand the development of 3D-printed sensors for water monitoring (Table 1).

### 3.1. Development of Sensors’ Platform Using 3D Printing

**Figure 3:**
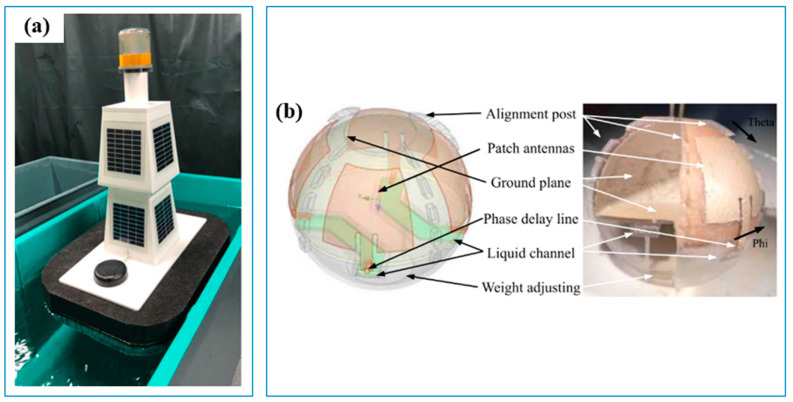
Three-dimensional-printed floating water quality measurement platform. (**a**) Floating water quality measurement system [48]. (**b**) Smart floating ball [15].

Three-dimensional printing has great advantages in fabricating complicated platforms supporting sensors’ operation in water monitoring. For instance, to detect the changes in water quality and avoid interference during manual collection, Kinar and Brinkmann [48] used FDM 3D printing to make a floating water-quality measurement platform using acrylonitrile styrene acrylate. This floating platform did not capsize during strong water turbulence caused by the wave. It could host multiple sensors to measure turbidity, total dissolved solids (TDS) and water temperature (Figure 3a). Compared to traditional manufacturing, this 3D-printed platform demonstrated great potential to create a low-cost network of sensors monitoring valuable data for the predictions of water quality. Notably, different designs using 3D printing technologies with similar purposes were also reported by Su et al. [15]. For example, an SLA 3D printer was used to manufacture a smart floating ball sensor with a complex structure and a metallization stamp for oil leakage detection (Figure 3b). Such a sensor is nearly impossible to be fabricated using traditional methods, but with 3D printing technology, they can be easily manufactured, and even modified to extend their applications for water quality detection and forecast.

**Figure 4:**
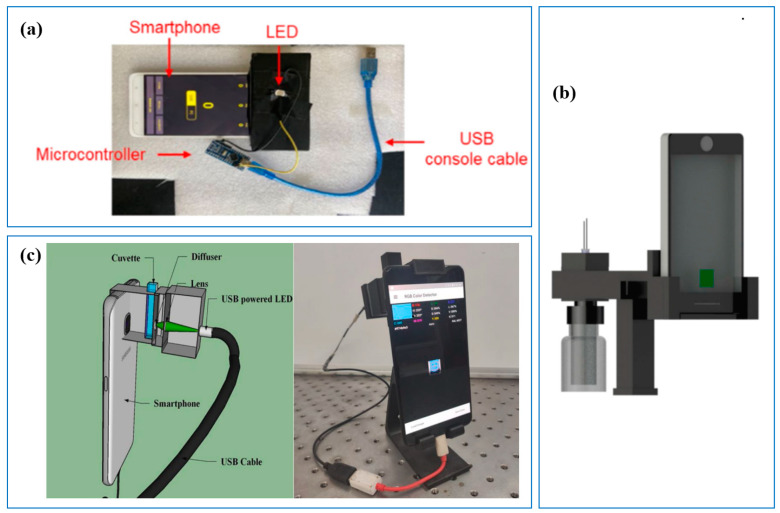
Three-dimensional-printed sensors’ accessories in the use of smartphones. (**a**) Three-dimensional-printed platform with smartphone ambient light sensor [43]; (**b**) Three-dimensional-printed determination platform [44]; (**c**) Schematic diagram and photograph of sensor [45].

The 3D-printed platform can also be used to compact all components into one system, making the sensor system operate easily and add functions conveniently, which results in enhanced sensitivity and efficiency. For instance, to use the light sensor of the smartphone, Gul et al. [43] designed a 3D-printed platform (Figure 4a) using the FFF method to accommodate different sample containers (e.g., cuvettes and membrane discs) and assay components (e.g., smartphones). This compact sensor system successfully detected the toxicant of 1,3-dichloro-2-propanol (1,3-DCP) in water, with a recovery rate of 101.95–109.70%. Similar studies were also carried out by Leal et al. [44] and Das et al. [45]. Using a 3D-printed platform with a smartphone, they developed an in situ aqueous sulfide sensor (Figure 4b) and a phosphate sensor (Figure 4c).

### 3.2. Development of Sensor’s Cell Using 3D Printing

**Figure 5:**
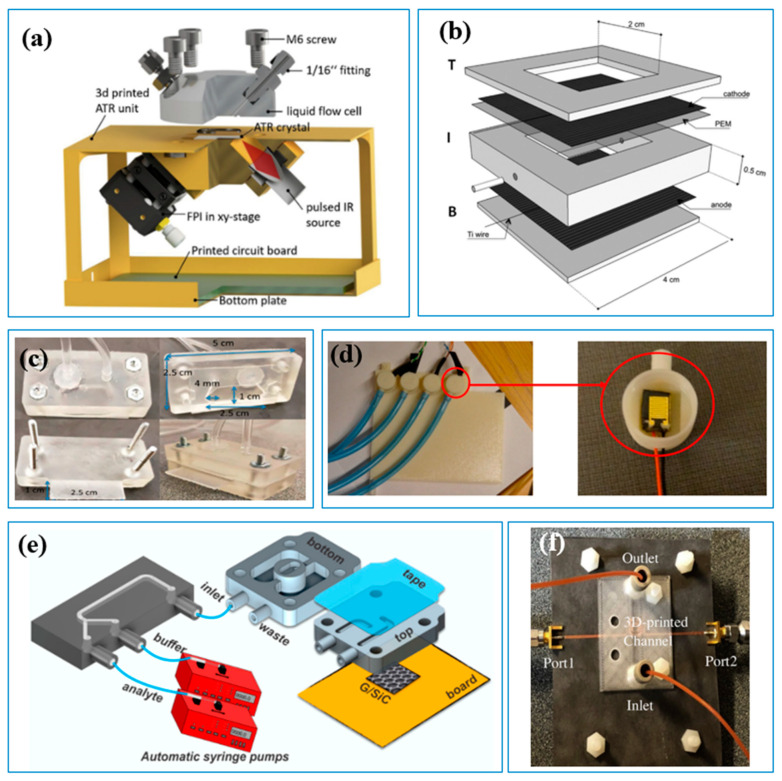
Three-dimensional-printed cells to improve sensors’ performance. (**a**) Three-dimensional-printed mini-spectrometer [42]; (**b**) Layer-by-layer 3D-printed miniature microbial fuel cell [63]. (**c**) Three-dimensional-printed flow cell [18]; (**d**) Three-dimensional-printed interface (right) and a pH sensor fitted into the interface (left) [11]; (**e**) Schematic of the sensing platform [49]; (**f**) Three-dimensional-printed microfluidic channel [50].

Three-dimensional printing has been widely used to fabricate sensor cells to improve design flexibility, modify the sensor’s geometry, increase the speed of the sensor response, increase sensors’ efficiency, prevent interference and lower the cost. Using the traditional manufacturing method would be very costly and time-consuming with many limitations in testing the researcher’s designs. In early studies, Bhattacharjee et al. [16] used FMD 3D print technology to print the cell of a water hardness sensor. The cell can not only hold the water sample but also has two LED housings and two photodiode housings. A more complicated sensor cell was fabricated by Baumgartner et al. [42]. They combined other components with 3D-printed units and created a pocket-sized 3D-printed nitrate sensor in water (Figure 5a). Similarly, Wong, et al. [59] made sensor casings by 3D printing. Microbial fuel cell (MFC)-based sensors are increasingly popular to monitor water quality, such as chemical oxygen demand (COD) and toxicants [60,61,62]. However, one of the significant drawbacks of MFC-based sensors is the slow response time, which is the time required to reach 95% of the steady-state electrical signals [51,60,63]. Three-dimensional printing with a fused filament fabrication method was employed to fabricate polycarbonate acrylonitrile butadiene styrene as the cell of the MFC sensor. Additionally, the cell was printed layer by layer to successfully incorporate the sensing elements and proton exchange membrane to prevent the potential water leak during the test. With the assistance of FFF/FDM technology, the sensor chamber volume was successfully reduced to 2 mL, which was only 1/6–1/30 of the previous typical MFC sensors manually fabricated (Figure 5b) [63,64]. During the test of COD in water samples, the response time was 2.8 min, which was sharply shorter than the 30–132 min of response time required in previous studies [63,64,65]. Additionally, this miniature sensor can also successfully detect the presence of cadmium in water with high sensitivity and a low detection limit.

The 3D-printed cell can improve the flow condition for better sensing performance. For instance, to overcome the influence of pressure and flow rate fluctuation on continuous online water-quality monitoring, Banna et al. [11] developed a conduit for miniaturized sensors using FDM 3D printing technology (Figure 5c), which successfully detected the pH and conductivity in water samples while avoiding interference from water flow rate and ambient temperature. To enhance the interaction between the sensing surface and liquid sample as well as provide high sensitivity and resolution, Santangelo et al. [49] developed a 3D-printed microfluidic lab-on-a-chip for the detection of heavy metals (Figure 5d), and Mohammadi et al. [50] developed a 3D-printed microfluidic channel for organic matters’ sensor, which can detect glucose concentrations as low as 46.7 mg L^−1^ (Figure 5e). Three-dimensional printing combined with numerical tools is a cost-effective approach to improve sensor cell geometry and enhance sensor performance. Zhao et al., [18] used computational fluid dynamic (CFD) simulation to optimize the detection parameters and then used the SLA 3D printer to manufacture the flow cell for a heavy-metal ion sensor, which efficiently enhanced the sensitivity of the sensor (Figure 5f). A similar approach was also conducted in the development of pH and conductivity sensors [11]. 

**Figure 6:**
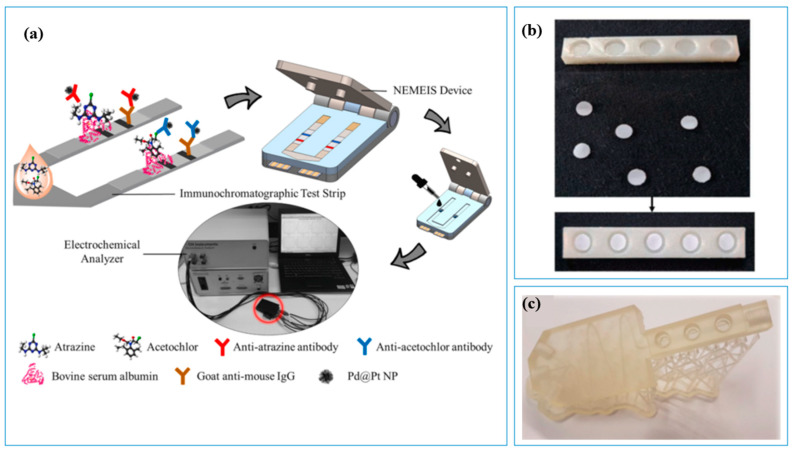
Three-dimensional-printed sensor platforms for muti-component analysis. (**a**) Electrochemical system [46]; (**b**) Three-dimensional-printed support with paper-based sensor [47]; (**c**) Three-dimensional-printed flow manifold [13].

With the assistance of 3D printing, it is convenient to develop a sensor array that can combine several sensors and simultaneously test different parameters in water. For example, Ruan et al. [46] developed a 3D-printed platform, which can detect atrazine and acetochlor at the same time (Figure 6a). Similarly, Debosz et al. [13] printed the flow manifold (Figure 6c), which can not only integrate several electrodes for simultaneously multi-component analysis, but also miniaturize electrodes. For multi-samples, Rajasulochana et al. [47] fabricated a 3D-printed platform to simultaneously test nitrite in several water samples (Figure 6b).

### 3.3. Development of Sensors’ Electrodes Using 3D Printing

**Figure 7:**
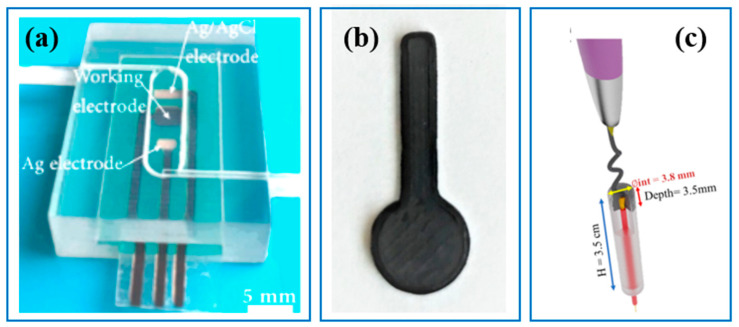
Three-dimensional-printed electrode for sensors. (**a**) Microfluidic with the device [17]; (**b**) Three-dimensional-printed electrode [52]; (**c**) Three-dimensional pen introducing conductive filament as the working electrode [54].

Three-dimensional printing technology also has great flexibility to make sensor electrodes, which are key sensing components. An early example of using 3D printing technology to fabricate sensor electrodes was shown by Hong et al. [17]. They used SLA 3D printing technology to manufacture the microfluidic cell and used screen-printing technology to make the flexible electrode. After modification, a real-time heavy metal ions sensor was successfully developed (Figure 7a). The limit of detection was 0.52 µg/L, which compares well with other sensors made by screen-printing technology. Additionally, the sensor showed high accuracy in comparison to commercial, electrothermal atomic-absorption spectrometry. This research demonstrates the great promise and extraordinary freedom of 3D printing for sensor manufacturing. Following this successful case study, Joyti et al. [52] used FDM 3D printing technology and carbon-loaded PLA material to make 3D-printed electrodes for the detection of chlorophenols and nitrophenols. They also found that their 3D-printed electrodes can be used for organophosphates (OPs) detections. They reported that a lot of effort has been put into traditional sensor-development methods but with limited outcomes. Their research-proven 3D printing technologies in sensor production has great advantages, including wide material choice, standardization and mass production (Figure 7b) [53]. With carbon-loaded conductive PLA material, Joao et al. [54] used a 3D printing pen to make an electrode manual for an on-site lead and copper concentration sensor (Figure 7c).

### 3.4. Development of all-3D-Printed Sensors

**Figure 8:**
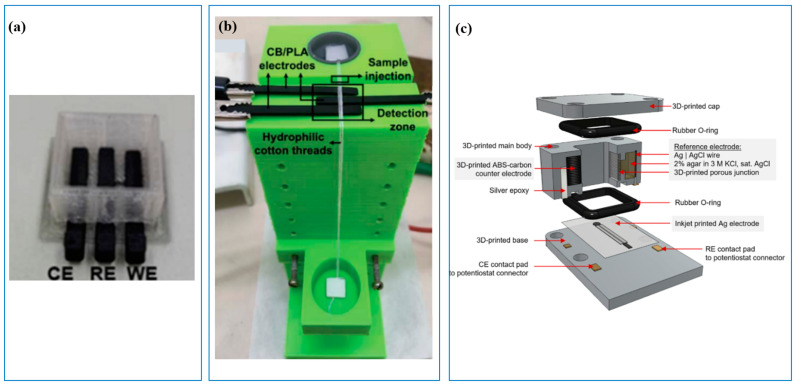
All-3D-printed sensors. (**a**) Three-dimensional-printed sensor for Hg(II) determination [55]; (**b**) Three-dimensional-printed sensor for nitrite sensor [56]; (**c**) Cross-section view of 3D-printed electrochemical cell for nitrate analysis [58].

With the successful applications of 3D printing technology fabricating each component of sensors, some researchers have started to explore the fabrication of all-3D-printed sensors for water analysis. In 2020, Katseli et al. [55] developed an integrated miniature all-3D-printed sensor for the determination of Hg(II) in bottled water and fish oil. They used FDM 3D-printing technology with different materials to print the mini vessel (PLA) and electrode (carbon-loaded PLA) (Figure 8a). After electrode surface modification, the sensor was ready for use. Using similar carbon-loaded PLA electrode materials, Carvalho et al. [56] and Pal et al. [57] developed all-3D-printed sensors for nitrate and nitrite (Figure 8b), respectively. In addition, for the nitrate sensor, Sibug-Torres et al. [58] used inkjet-printing to print the Ag (IJP-Ag) as the working electrode, FDM 3D-printed cell (ABS), and counter electrodes (carbon-loaded ABS) (Figure 8c). Using FDM technology, a capacitance-based water pressure sensor was also successfully developed [66]. The sensing section consisted of two plates, one fixed while the other movable. The movable plate deflected, and capacitance changed when the pressure was applied. The maximum sensitivity of this sensor was 5.36 × 10^−2^ pF/hPa, which was much higher than the previously reported sensor (1.4 × 10^−2^ pF/hPa) [67]. Combined with the numerical model, the overall cost of the developed sensor was substantially reduced to around EUR 1.5, which was only around 2.5–8.3% of typical commercial prices of a pressure sensor. Similar progress was also reported in other pressure and thermal sensors fabricated using 3D printing [68,69,70]. It should be noted that all-3D-printed sensors are still at the proof-of-concept stage, and the majority of them use FDM 3D printing technologies and carbon-loaded polymer materials.

## 4. Outlook

In this review, we summarized the development history and market share of typical 3D-printing technologies and their applications in sensor development for water monitoring and analysis. Before the application of 3D printing (additive manufacturing), sensors were fabricated using traditional manufacturing methods, including subtractive manufacturing (also known as machining, such as turning, boring, and milling) and forming or even manually. These methods could be costly, hard to use, limited in geometry and very unfriendly for prototype production in small quantities [71], which has restricted sensors research.

As a powerful tool, compared to the traditional manufacturing method, 3D printing clearly showed multiple unique advantages. It provides huge flexibility for sensor design and manufacturing. Facilitated by 3D printing, researchers are able to design and fabricate very complex structures that are very hard or even impossible to be fabricated using traditional manufacturing methods, such as a floating sphere [15] and sensor array [47]. It is also a cost-effective approach for sensor modification and upgrades. With the assistance of 3D printing technology, researchers can validate their ideas, calculations, and/or simulations quicker and cheaper than with traditional manufacturing methods. Currently, 3D printing technology is experiencing rapid development. Many novel methods and improved materials are incorporated into this technology. This will definitely benefit the development of 3D-printed sensors. 

To date, it has been less than 10 years since 3D printing was first applied to water sensors’ development. Whilst this technology substantially promotes sensors’ development, there are still some major drawbacks in current studies that should be noted in future research.

These sensors developed by 3D printing are not sufficient to comprehensively monitor and assess water quality. To monitor water quality, there is a wide range of important parameters to be characterized, including bacteria (e.g., *E. coli*), ammonia, alkalinity and acidity and chloride [9]. However, to the best of our knowledge, there are no 3D-printed sensors developed to monitor those crucial parameters. Evidently, more research should be focused on those missing parameters related to water quality.

The current research on sensors does not realize the full potential of 3D printing technology. The majority of researchers use FDM/FFF 3D-printing technology (Table 1), which is also the most common 3D printing technology nowadays (Figure 1). It provides a fast, cost-effective and easy-to-use manufacturing method for researchers. However, it still has many disadvantages, such as limited choice of materials (e.g., polymer or carbon-loaded polymer). There are already dozens of mature 3D-printing technologies, with extensive material choices, including metals, ceramics and even gels. Further study should explore and compare different 3D printing techniques with different materials and expand possibilities in water analysis sensors.

It is necessary to validate and improve the long-term stability of 3D-printed sensors in real water monitoring and prevent the potential contamination caused by the sensors. Most of the reported sensors are prototypes, which have limited real water monitoring. Currently, PLA is a very popular material used in sensor development [72]. However, it is well known that PLA is a biodegradable material, which can be damaged by UV exposure, bacteria and hydrolysis degradation [73]. Given that these sensors are repeatedly exposed to harsh conditions (e.g., UV, water, and mechanical shaking), the functional stability of these sensors in the field is clearly needed. Additionally, the degradation of these polymer-based sensors may release high-level pollutants (e.g., plastic additives [74,75] and microplastics [76,77,78]) to the monitoring water body. Currently, microplastics and organic additives sourcing from plastics are a global concern due to their potential threat to environmental and human health [78,79,80]. Those should also be taken into consideration during the life cycle assessment of 3D-printed sensors.

Finally, it is urgent to establish a standard report protocol to ensure the research results are comparable. To date, most studies in the area of 3D-printed sensors are at the proof-of-concept stage, and their reported parameters are heavily dependent on authors’ preference, which leads to the loss of some crucial information. For instance, the majority of them did not compare the 3D-printed sensors with traditionally manufactured sensors, with only a few making the comparison [55,66]. The reported test parameters also varied case by case, which leads to a comparison between different cases being extremely difficult, if not impossible. Evidently, a standard report protocol including necessary parameters (such as sensitivity, accuracy, detection range, response time, and sensors’ comparison) is desperately required for the progress of 3D-printed sensors.

## Figures and Tables

**Table 1:** Summary of 3D printing applications in sensors’ development.

3D-Printed Component	3D Printing Technology	Materials	Main Functions of 3D-Printed Component	Detection Object	Reference
Platform	FFF/FDM	ABS *	Structural component	Water hardness	[16]
	FFF/FDM	PLA *	Structural component	Nitrate	[42]
	FFF/FDM	-	Platform	1,3-DCP	[43]
	FFF/FDM	-	Platform	Sulfide	[44]
	FFF/FDM	-	Platform	Phosphate	[45]
	FFF/FDM	PLA *	Platform	Atrazine and acetochlor	[46]
	FFF/FDM	PLA *	Platform	Nitrite	[47]
	SLA	Clear resin	Flow manifold	Potassium, sodium, calcium and chloride	[13]
	FFF/FDM	Acrylonitrile styrene acrylate	Floating platform	Turbidity, total dissolved solids, and water temperature	[48]
	SLA	Clear resin	Floating platform	Potential water pollution, such as crude oil leakage	[15]
Cell	SLA	Clear resin	Flow cell	Heavy metal ions	[18]
	FFF/FDM	ABS *	Conduit	Water quality	[11]
	SLA	Resin Clear Type 02	Microfluidic lab-on-a-chip	Heavy metal ions	[49]
	FFF/FDM	Copolyester	Microfluidic channel	Organic matters	[50]
	FFF/FDM	PC/ABS *	Sensor cell; glue all components; seal sensor	Chemical oxygen demand; cadmium	[51]
Electrode	Screen printing	-	Microfluidic with electrode	Heavy metal ions	[17]
	FFF/FDM	Carbon-loaded PLA *	Electrode	Chlorophenols and nitrophenols	[52]
	FFF/FDM	Carbon-loaded PLA *	Electrode	OPs	[53]
	3D printing pen	Carbon-loaded PLA *	Working electrode	Lead and copper	[54]
All-3D-printed sensors	FFF/FDM	Carbon-loaded PLA *	Electrochemical cell	Hg(II)	[55]
	FFF/FDM	Carbon-loaded PLA *	Embedded electrochemical detector	Nitrate	[56]
	FFF/FDM	Carbon-loaded PLA *	Miniaturized electrochemical sensor	Nitrite	[57]
	Inject and FFF/FDM	Ag and carbon-loaded ABS *	Electrochemical cell	Nitrate	[58]

* ABS: acrylonitrile butadiene styrene; PC-polycarbonate; PLA-polylactic acid.

## Data Availability

All the raw data are available by contacting L.X.
